# Patient satisfaction with general practice in Scotland 2011/12 to 2021/22

**DOI:** 10.1371/journal.pone.0322095

**Published:** 2025-04-30

**Authors:** David Henderson, Eddie Donaghy, Kieran Sweeney, Bruce Guthrie, Andrew G. H. Thompson, Harry H. X. Wang, Stewart W. Mercer

**Affiliations:** 1 College of Medicine and Veterinary Medicine, Usher Institute, University of Edinburgh, Edinburgh, United Kingdom,; 2 School of Social and Political Science, University of Edinburgh, Edinburgh, United Kingdom,; 3 School of Public Health, Sun Yat-Sen university, Guangzhou, China; UCL: University College London, UNITED KINGDOM OF GREAT BRITAIN AND NORTHERN IRELAND

## Abstract

**Background:**

The Scottish Government introduced the first phase of a new General Practice (GP) contract in 2018, aiming to transform primary care and address health inequalities. However, the impact of these changes on patient satisfaction is unclear.

**Aim:**

To assess temporal changes in overall patient satisfaction, and satisfaction with access and consultation quality, in general practice between 2011/12 and 2021/22, focusing on disparities across sociodemographic groups.

**Design and Setting:**

Analysis of biennial national Health and Care Experience (HACE) survey data from patients in Scotland, spanning six survey waves.

**Methods:**

Descriptive analyses of trends in patient satisfaction, access, and consultation quality. Disparities in deprivation were measured by the Relative Index of Inequality (RII).

**Results:**

Overall patient satisfaction with general practice declined significantly over the 10 years, with mean positive scores dropping from 90.1% in 2011/12 to 70.5% in 2021/22. Satisfaction was lower in patients living in more deprived areas at all time points, and the gap between the most and least deprived populations widened over time, with the RII increasing from 1.05 (95% CI 1.04–1.06) in 2011/12 to 1.12 (95% CI 1.08–1.15) in 2021/22. Overall satisfaction and access satisfaction had the most pronounced declines, especially among younger patients and those with multiple long-term conditions. In contrast, consultation quality measures (whether patients felt listened to and had enough time during consultations) remained largely stable with only slight declines observed.

**Conclusion:**

Satisfaction overall, and with access to GP consultations, steadily declined between 2011/12 and 2021/22, with a more pronounced decrease following the COVID-19 pandemic, particularly among the most deprived and complex patients. Although the new GP contract was introduced during this period, it does not appear to have significantly impacted these downward trends. These findings highlight the need for focused efforts to improve patient satisfaction, especially in disadvantaged populations, as the contract evolves.

## Introduction

The landscape of primary care delivery is undergoing profound transformation internationally driven by population ageing, changing patterns of disease, and the increasing complexity of patient needs. [1] The ageing UK population, accompanied by a rising prevalence of multimorbidity places unprecedented pressure on primary care services. [2] In response to these challenges, national and devolved governments across the UK have implemented significant policy changes aimed at optimizing care services. [3–5] In the UK, primary care is principally delivered through community-based General Practitioner (GP) practices, which serve as the first point of contact for patients and are supported by a multidisciplinary team that includes nurses, pharmacists, and allied health professionals to provide comprehensive, continuous care.

In April 2018, a new General Medical Services (GMS) contract was introduced in Scotland, representing the first GP contract developed distinctly from the rest of the UK. This marked a significant milestone in the evolution of healthcare services. [6,7] The formal contract was preceded by abolishment of the pay-for-performance Quality and Outcomes Framework (QOF) and establishment of GP clusters (small numbers of local GP practices working together to meet local needs) in 2016, followed by a substantial expansion of the multidisciplinary team (MDT) in primary care. [8] The new contract aimed to address the evolving needs of an ageing population, health inequalities, and the quality of care provided by general practices. [9]

The ability to mitigate health inequalities, through timely access and quality, means that healthcare itself is a social determinant of health, [10] yet health inequalities remain wider in Scotland than anywhere else in western Europe. [11] The inverse care law articulated by Julian Tudor-Hart over 50 years ago posits that those with the greatest need often have the least access to good healthcare,[12] and this phenomenon remains relevant in many countries, including Scotland. [13,14]

Against this backdrop, the evaluation of patient satisfaction, as well as patient views on access to and quality of primary care becomes a crucial lens through which to assess the impact of healthcare reforms and understand the disparities in service delivery. [15–17] This is particularly important in primary care, where people mainly self-initiate their interaction with the health care system.

Our study investigates changes in patient satisfaction with general practice and patient views on access and quality of care in Scotland from 2011/12–2021/22, covering the period immediately preceding and following the abolition of QOF and the introduction of GP clusters in 2016 as well as the formal introduction of the GP contract in April 2018, including before and after the COVID-19 pandemic, with the aim of assessing temporal changes in patient satisfaction in general practice over a decade, between 2012 and 2022.

## Methods

### Study design

We conducted a secondary analysis of data from the Scottish Government (SG)-collected Health and Care Experience Survey (HACE) linked to a population register to investigate overall satisfaction with general practice among people living in Scotland between 2011/12 and 2021/22. We aimed to assess changes in satisfaction and patient perception of access and quality over time, taking into account various demographic factors including age, sex, self-reported health, deprivation status, and urban/rural classification. Additionally, the relative index of inequality (RII) was calculated to measure disparities in satisfaction between those living in the most and least deprived areas of Scotland with results stratified by the above sociodemographic variables.

### Data sources

HACE, the primary data source, is collected by the SG every two years and asks about adult’s experience of use and access of their GP practice, aspects of care and support provided by local authorities, and caring responsibilities. [18] Requests to complete the survey, either by post or online are sent to a random sample of people aged 17 or over and registered with a GP in Scotland aiming to receive a minimum number of responses from every GP practice. Between 537,924 and 711,159 survey requests were issued between 2011/12 and 2021/22 with overall response rate varying between 16% and 24%. In all waves, data were collected between October and February, although the exact timing varied somewhat. Complete details of the sampling strategy and survey response can be found in the technical reports of each wave of the survey. [18]

Published survey results include limited demographic data. To obtain a broader range of detail we obtained pseudonymised, unweighted survey responses linked to the Community Health Index (CHI) population spine which lists all patients registered with a GP in Scotland. [19] We requested demographic data for all HACE respondents aged 20 and over. As HACE is sampled from CHI, there was a 100% match rate.

### Variables

Throughout the six waves of our study spanning from 2011/12–2021/22, the survey underwent certain modifications in question formulation and wording. However, core questions pertaining to satisfaction, access, and consultation quality remained consistent enough to permit meaningful comparisons. Notably, questions concerning satisfaction and access retained their core structure across all survey waves. Regarding consultation quality, there was a shift in question wording between early and later survey waves. Initially framed as, “Thinking of the last time you saw a doctor at your GP surgery, how much do you agree or disagree with each of the following?” the wording transitioned to, “Thinking about that healthcare professional [previous question], how much do you agree or disagree with the following statements?” in the last three survey waves. This adjustment reflected an enhancement aimed at capturing respondents’ reflections on their most recent consultation experience at the practice, inclusive of interactions with non-medical members of the multidisciplinary team. Despite these nuanced changes, the fundamental themes and objectives underlying the consultation quality questions remained consistent throughout the study period. In the subsequent waves of this study, we specifically focused on responses pertaining to GP consultations, thereby ensuring the comparability of data across survey waves. Specific variables used in the analysis were as following:

1. *Outcome 1-Overall satisfaction: “Overall, how would you rate the care provided by your GP surgery/practice?”* Potential responses were: Excellent, Good, Fair, Poor, or Very Poor which we collapsed into positive (Excellent or Good), neutral (Fair) or negative (Poor or Very Poor) categories and calculated the mean percentage of positive responses at GP practice-level and across sociodemographic characteristics.
2. *Outcome 2-Access*: “Overall, how would you rate getting to see a doctor in your practice/surgery?” Responses and recoding as above.

*Outcome 3-Consultation quality*: “Thinking of the last time you saw a doctor at your GP surgery, how much do you agree or disagree with each of the following?”/ “Thinking about that healthcare professional [previous question], how much do you agree or disagree with the following statements?”

a. The doctor listened to me/ I was listened to
b. I had enough time with the doctor/ I was given enough time

Potential responses were: “Strongly agree”, “Agree”, “Neither Agree nor Disagree”, “Disagree”, “Strongly Disagree” which we collapsed into positive (Strongly agree or Agree), neutral (Neither agree nor Disagree) and negative (Disagree or Strongly disagree) categories and calculated the mean percentage of positive responses at GP practice-level and across sociodemographic characteristics.

1. *Sociodemographic variables*: The following demographic variables were considered:
  *Age*: Sourced from CHI register and categorised into broad age groups.
  *Sex*: Sourced from CHI register and categorised as male or female.
  *Multimorbidity*: Sourced from HACE with count of positive responses to the question *“Do you have any of the following? 1. Deafness or severe hearing impairment 2. Blindness or severe vision impairment 3. A physical disability 4. Chronic pain lasting at least 3 months 5. A mental health condition 6. A learning disability 7. Another long-term condition”*.
  *Deprivation decile/quintile*: Sourced from CHI register. Scottish Index of Multiple Deprivation 2020 (SIMD) [20] decile of residence.
  *Urban/Rural classification:* Sourced from CHI register. Scottish Government Urban Rural Classification 2020 [21] category of residence dichotomised into accessible and remote.

### Statistical analysis

The statistical analysis was designed to explore temporal trends and disparities in patient satisfaction, access, and consultation quality over time, with a focus on sociodemographic factors. Descriptive statistics were first employed to summarise the characteristics of HACE survey respondents and to chart overall trends in satisfaction across the study period. The analysis examined the mean percentage of positive responses for satisfaction, access, the extent to which patients felt listened to, and whether they had enough time during consultations. These outcomes were analysed according to key sociodemographic variables: age, sex, number of health issues, deprivation status, and urban/rural classification.

To assess whether disparity in mean overall satisfaction increased over time we calculated the RII for each year and stratified by sociodemographic variables. We used the RII rather than the Slope Index of Inequality (SII) to negate the sensitivity of the latter measure to changes in mean outcome over time [22]. We used the *phe_sii* function from the R Package *PHEindicatormethods* [23] where the RII is calculated by taking the ratio of the Slope Index of inequality (SII) in the most and least deprived deciles.

### Ethical considerations

Approval for this study was granted by the University of Edinburgh Medical School Research Ethics Committee (Ref: 21-EMREC-023) and Public Benefit and Privacy Panel for Health and Social Care (PBPP) (Ref: 2122–0004). Approval from PBPP is required to ensure all data remains pseudonymised and data released from secure environments is subject to statistical disclosure control. Full details of secure data access process have been described elsewhere. [24] Data was processed under Article 6(1)(e) and Article 9(2)(j) of the EU General Data Protection Regulation and a Data sharing agreement was signed between the Scottish Government and the University of Edinburgh. Consent for the use of pseudonymised data for research is covered in the online privacy notice for the Health and Care Experience survey. [25] Data was first accessed on 13/12/2022 and was available for analysis until 31/03/2024.

## Results

After data cleaning there was a total of 779,504 responses to HACE over the six bi-annual waves 2011/12–2021/22 included in the analysis ranging from 109,644 responses (2015/16) to 142,724 (2011/12) per wave (Table 1). There were fewer respondents in 2013/14 and 2015/16 compared to other years (representing 14.2% and 14.1% of overall responses, respectively). More women than men responded in all years (56.9% v 43.1% overall) and responses in age-bands were broadly similar in each wave. There were more respondents from SIMD deciles 4–9 than others with 8.5% of responses coming from people living in the most deprived decile 1 in total, and 9.4% coming from the least deprived decile 10. Between 16.1% (2017/18) and 18.8% (2021/22) of respondents selected two or more health issues from the list provided in HACE. The percentage of respondents from each level of Urban/Rural category was consistent over time with approximately 84.8% and 15.2% responding from Accessible and Remote areas, respectively.

**Table 1:** Characteristics of HACE respondents by year.

	Total	2011/12	2013/14	2015/16	2017/18	2019/20	2021/22
**Total N (%)**	**779504**	**142724 (18.3)**	**110697 (14.2)**	**109644 (14.1)**	**131029 (16.8)**	**157445 (20.2)**	**127965 (16.4)**
**Sex**							
Male	335968 (43.1)	60175 (42.2)	47838 (43.2)	47090 (42.9)	57022 (43.5)	68145 (43.3)	55698 (43.5)
Female	443536 (56.9)	82549 (57.8)	62859 (56.8)	62554 (57.1)	74007 (56.5)	89300 (56.7)	72267 (56.5)
**Age Band**							
20-44	155937 (20.0)	33049 (23.2)	22281 (20.1)	19852 (18.1)	22658 (17.3)	32529 (20.7)	25568 (20.0)
45-69	409331 (52.5)	75593 (53.0)	59802 (54.0)	59689 (54.4)	68824 (52.5)	80273 (51.0)	65150 (50.9)
70+	214236 (27.5)	34082 (23.9)	28614 (25.8)	30103 (27.5)	39547 (30.2)	44643 (28.4)	37247 (29.1)
**SIMD Decile**							
1 - Most Deprived	66067 (8.5)	12726 (8.9)	9660 (8.7)	9012 (8.2)	10906 (8.3)	13239 (8.4)	10524 (8.2)
2	64870 (8.3)	12207 (8.6)	9309 (8.4)	8737 (8.0)	10853 (8.3)	13428 (8.5)	10336 (8.1)
3	68581 (8.8)	12788 (9.0)	9849 (8.9)	9487 (8.7)	11607 (8.9)	13924 (8.8)	10926 (8.5)
4	75836 (9.7)	14449 (10.1)	10664 (9.6)	10795 (9.8)	12733 (9.7)	15045 (9.6)	12150 (9.5)
5	90817 (11.7)	16888 (11.8)	12982 (11.7)	12923 (11.8)	15310 (11.7)	18011 (11.4)	14703 (11.5)
6	97152 (12.5)	17628 (12.4)	13687 (12.4)	13690 (12.5)	16612 (12.7)	19597 (12.4)	15938 (12.5)
7	85759 (11.0)	15223 (10.7)	12021 (10.9)	12188 (11.1)	14312 (10.9)	17603 (11.2)	14412 (11.3)
8	79531 (10.2)	13929 (9.8)	10986 (9.9)	11277 (10.3)	13273 (10.1)	16381 (10.4)	13685 (10.7)
9	77428 (9.9)	13464 (9.4)	11013 (9.9)	10953 (10.0)	12908 (9.9)	15680 (10.0)	13410 (10.5)
10 - Least Deprived	73463 (9.4)	13422 (9.4)	10526 (9.5)	10582 (9.7)	12515 (9.6)	14537 (9.2)	11881 (9.3)
**N Conditions**							
Less than two	637984 (81.8)	116212 (81.4)	89996 (81.3)	89083 (81.2)	109888 (83.9)	128954 (81.9)	103851 (81.2)
Two or more	141520 (18.2)	26512 (18.6)	20701 (18.7)	20561 (18.8)	21141 (16.1)	28491 (18.1)	24114 (18.8)
**Accessible/Rural status**							
Accessible	661393 (84.8)	120045 (84.1)	93851 (84.8)	92432 (84.3)	111179 (84.9)	134681 (85.5)	109205 (85.3)
Remote	118111 (15.2)	22679 (15.9)	16846 (15.2)	17212 (15.7)	19850 (15.1)	22764 (14.5)	18760 (14.7)
**Overall Satisfaction**							
Negative	29204 (4.3)	2618 (2.0)	2233 (2.3)	2463 (2.5)	3816 (3.4)	6521 (4.8)	11553 (11.5)
Neutral	75322 (11.2)	10169 (7.9)	8719 (9.0)	8578 (8.8)	12206 (10.8)	17699 (13.1)	17951 (17.9)
Positive	568514 (84.5)	116531 (90.1)	86329 (88.7)	86821 (88.7)	97470 (85.9)	110803 (82.1)	70560 (70.5)
**Getting to see the doctor**							
Negative	67358 (10.2)	7877 (6.2)	7022 (7.2)	7837 (8.0)	13169 (11.6)	16250 (12.1)	15203 (16.4)
Neutral	104018 (15.7)	17446 (13.7)	14622 (15.1)	14934 (15.3)	18242 (16.1)	21497 (16.0)	17277 (18.6)
Positive	491533 (74.1)	102206 (80.1)	75332 (77.7)	74589 (76.6)	82219 (72.4)	96727 (71.9)	60460 (65.1)
**The doctor listened to me**							
Negative	10995 (2.1)	1855 (1.6)	1384 (1.6)	1437 (1.7)	1449 (1.9)	2216 (2.4)	2654 (4.5)
Neutral	17332 (3.3)	3626 (3.1)	2472 (2.9)	2405 (2.8)	2614 (3.4)	3113 (3.3)	3102 (5.3)
Positive	492567 (94.6)	113276 (95.4)	82402 (95.5)	82325 (95.5)	73897 (94.8)	87871 (94.3)	52796 (90.2)
**I had enough time with the doctor**							
Negative	21704 (4.2)	4154 (3.5)	3286 (3.8)	3805 (4.4)	3078 (4.0)	3846 (4.2)	3535 (6.2)
Neutral	29849 (5.8)	5899 (5.0)	4765 (5.5)	5086 (5.9)	4351 (5.7)	5119 (5.5)	4629 (8.1)
Positive	464879 (90)	108228 (91.5)	77975 (90.6)	76917 (89.6)	69533 (90.3)	83431 (90.3)	48795 (85.7)

The percentage of positive responses to satisfaction questions showed a gradual decline between 2011/12 and 2019/20, with overall satisfaction dropping from 90.1% to 82.1% and access falling from 80.1% to 71.9%. The decline became more pronounced between 2019/20 and 2021/22, with satisfaction further decreasing to 70.5% and access to 65.1%. Consultation quality showed a more modest reduction during the same period. The percentage of respondents who felt their doctor listened to them fell slightly from 95.4% in 2011/12 to 94.3% in 2019/20, and those who felt they had enough time with their doctor decreased from 91.5% to 90.3%. However, between 2019/20 and 2021/22, more significant drops were observed, with positive responses for listening declining to 90.2% and for time spent with the doctor dropping to 85.7%. These results suggest that while overall satisfaction and access saw larger decreases, consultation quality also decreased, especially in the latter period.

**Fig 1:**
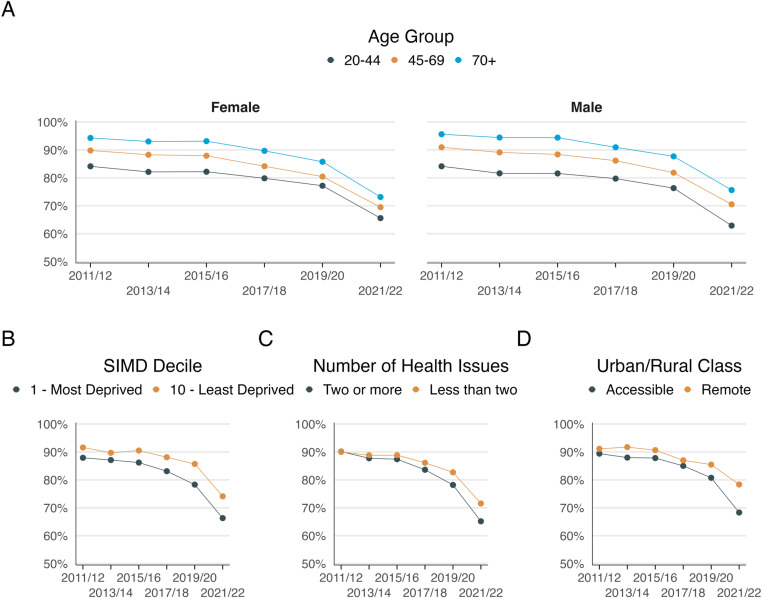
Percentage of positive responses to overall satisfaction 2011/12 by A) Age and sex B) SIMD decile C) Number of Health Issues and D) Urban/Rural Class.

**Fig 2:**
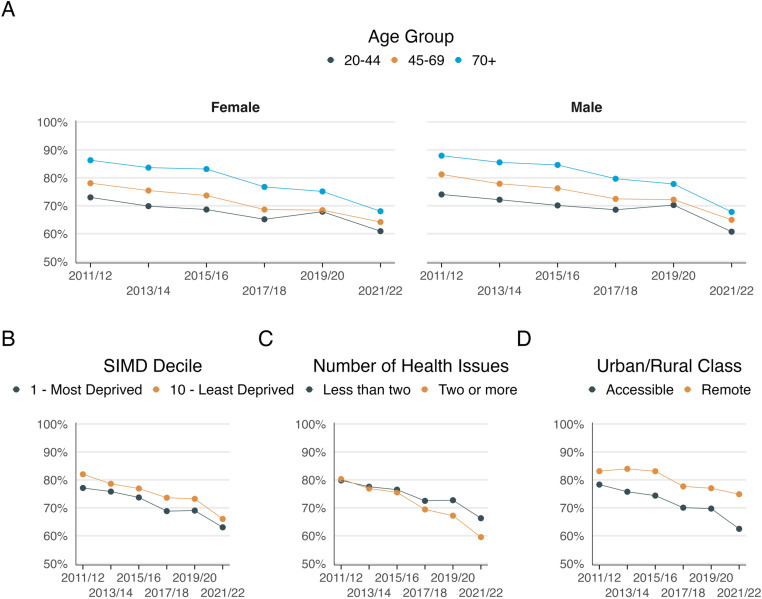
Percentage of positive responses to access question 2011/12 by A) Age and sex B) SIMD decile C) Number of Health Issues and D) Urban/Rural Class.

Figures 1 and 2, along with Supplementary Figures S1 and S2, display the overall mean percentage of positive responses to satisfaction, access, consultation listening, and time questions, each stratified by sociodemographic characteristics. The observed patterns generally followed the overall trends. Satisfaction and access saw the largest declines, particularly among respondents from the most deprived areas, those with multimorbidity, and individuals living in accessible areas. The decline in positive responses regarding consultation quality—whether patients felt listened to or had enough time during consultations—was more gradual but still pronounced among those in deprived areas and with multimorbidity. These findings suggest that declines were more severe across all measures for individuals in deprived areas and those with multimorbidity, even in consultation quality measures, which otherwise showed smaller decreases overall.

**Fig 3:**
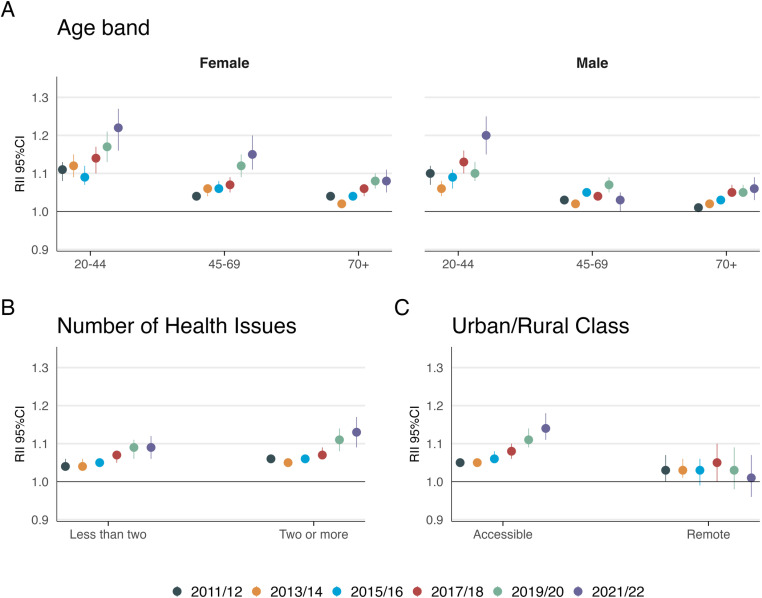
Relative Index of Inequality (RII) and 95% Confidence Interval (CI) for overall satisfaction between most a least deprived SIMD decile by A) Age and sex B) Number of Health Issues and c) Urban/Rural Class.

Examining the RII results, Supplementary Table S1 and Figure S3 illustrate disparities in all measures over time. The RII for overall satisfaction increased from 1.05 (95% CI 1.04–1.06) in 2011/12 to 1.12 (95% CI 1.08–1.15) in 2021/22, indicating a widening gap in satisfaction between the most and least deprived populations. This pattern was consistent across various sociodemographic factors, with the largest disparities observed in younger age groups and among respondents with multimorbidity (Figure 3 and Supplementary Tables S2-S4). In 2021/22, the highest RIIs were found among females aged 20–44 years (RII 1.22 [95% CI 1.16–1.27]), males in the same age group (RII 1.20 [95% CI 1.15–1.25]), and respondents living in accessible urban/rural areas (RII 1.14 [95% CI 1.11–1.18]).

**Fig 4:**
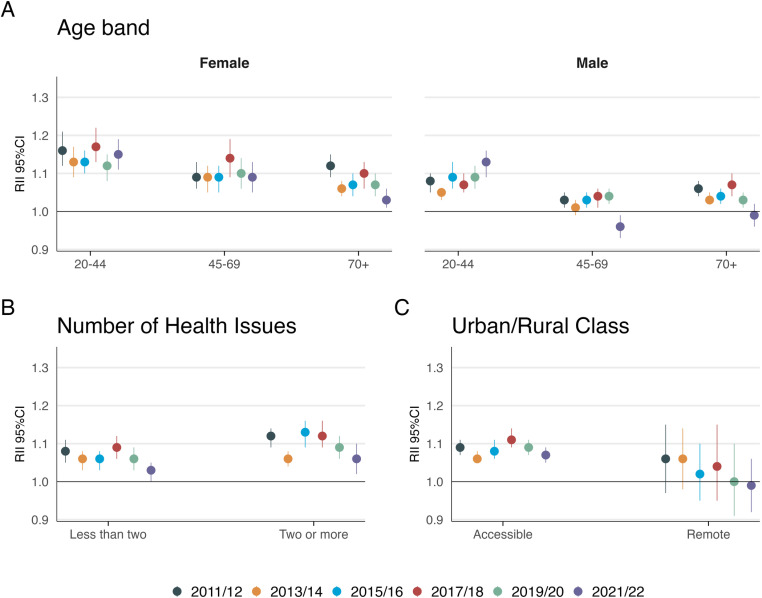
Relative Index of Inequality (RII) and 95% Confidence Interval (CI) for access question between most a least deprived SIMD decile by A) Age and sex B) Number of Health Issues and c) Urban/Rural Class.

The RII for access (Supplementary Table S1 and Figure S3) presented a more complex picture. While access satisfaction declined overall, the RII fluctuated throughout the study period and was slightly lower in 2021/22 compared to 2011/12, although the confidence intervals overlap (RII 1.05 [95% CI 1.02–1.08] vs. 1.09 [95% CI 1.06–1.11]). Despite this, disparities remained, with the largest gaps observed in younger age groups and those with multimorbidity (Figure 4 and Supplementary Tables S2-S4).

The RII for consultation quality also indicated increasing inequality over time. For the question on whether patients felt listened to, the RII rose from 1.02 (95% CI 1.02–1.03) in 2011/12 to 1.06 (95% CI 1.05–1.08) in 2021/22 (Supplementary Table S1 and Figure S3). This suggests emerging disparities between the most and least deprived populations, particularly among younger respondents and those with multimorbidity (Supplementary Figure S4 and Tables S2–S4).

Similarly, the RII for whether patients felt they had enough time during consultations increased from 1.02 (95% CI 1.01–1.03) in 2011/12 to 1.08 (95% CI 1.06–1.11) in 2021/22 (Supplementary Table S1 and Figure S3). This rise in inequality highlights growing disparities in consultation time, consistent with those observed in the listening responses. Similar patterns were noted across sociodemographic subgroups (Supplementary Figure S5 and Tables S2–S4), indicating that even in areas of care that were more resilient to decline, inequalities between the most and least deprived groups have widened over time.

## Discussion

### Summary

This analysis of overall patient satisfaction with general practice in Scotland between 2011/12 and 2021/22 reveals a significant decline in satisfaction and access over time, with mean positive scores for overall satisfaction dropping from 90.1% to 70.5%. The gap between the most and least deprived SIMD deciles widened notably, with the RII for overall satisfaction increasing from 1.05 (95% CI 1.04–1.06) in 2011/12 to 1.12 (95% CI 1.08–1.15) in 2021/22. This growing disparity was most pronounced among younger age groups and those with multimorbidity, who also experienced the greatest absolute declines in satisfaction and access to care.

Measures of consultation quality, specifically whether patients felt listened to and had enough time during their consultations, showed smaller declines, but the gap between the most and least deprived also widened over time. Although the decline in measures of consultation quality were smaller than for access, previous research has shown that consultation quality accounts for a much higher proportion of overall satisfaction than access. [26] Thus, even small declines in consultation quality may be of relevance, especially given the evidence that indicators of good interpersonal communication by GPs in the consultation is associated with higher patient enablement and better health outcomes. [27,28]

It should be noted that the introduction of the new Scottish GP contract in 2018 has not reversed these downward trends, highlighting the need for targeted efforts to address the widening inequalities in patient experience.

The sharp decline in satisfaction observed in the most recent wave of data (2021/22) is likely linked to the impact of the COVID-19 pandemic. During this period, healthcare services across Scotland and globally faced unprecedented strain, including restrictions on face-to-face consultations and disruptions to routine care. These challenges would have disproportionately affected access to GP services, explaining the steep drop in satisfaction scores between 2019/20 and 2021/22. However, it is important to acknowledge that the downward trend in satisfaction began well before the pandemic, with gradual declines observed throughout the study period. This suggests that while the pandemic likely exacerbated existing issues, the underlying drivers of dissatisfaction—particularly for deprived and multimorbid populations—were already in place and need to be addressed through long-term, structural changes to primary care delivery.

### Strengths and limitations

Our study benefits from several strengths, including the use of a Scottish Government-collected survey linked to a population register, which provides a comprehensive and representative dataset. The sampling design of HACE is based on GP practice lists and aims to receive a pre-specified number of responses from each practice based on list size [18]. The ability to stratify results by various sociodemographic factors allowed for a nuanced analysis of satisfaction trends. The calculation of the RII index facilitated the assessment of relative disparities, taking the overall distribution of the outcome across deprivation deciles into account.

However, the study has several limitations. Firstly, the reliance on self-reported satisfaction measures may introduce response bias, as individuals may have varying perceptions of what constitutes satisfactory healthcare. Whilst the sampling design ensured sufficient responses from each GP practice, there were variations in response across sociodemographic groups with proportionately fewer responses from men and those in younger age groups [18], although people sent the survey are asked to respond only if they have recently used the practice, so this may partly reflect varying primary care use. There were also proportionately fewer responses from those living in more deprived areas and urban areas [18], a common limitation to many surveys. Finally, the measure of multimorbidity in this study was derived from self-reported responses to a limited number of health conditions, which may not fully capture the complexity or extent of an individual’s health status. This method could lead to underreporting or misclassification of multimorbidity, as respondents may not recognize or disclose all relevant conditions

### Comparison with previous literature

In the most recent Health and Care Experience Survey 2023/24 [18], published since our analysis was conducted, satisfaction with general practice has somewhat stabilized, with 69% of respondents rating their overall experience as good or excellent, a slight increase from 67% in 2021/22 but still below pre-pandemic levels. Regarding specific outcomes, the 2023/24 survey reports on the time patients felt they were given and whether they were listened to, but these questions relate to the health professional the respondent saw, which may not necessarily have been a doctor, and thus cannot be directly compared with our findings.

The findings in this study confirm those from our related work with patients from a smaller number of GP practices in Scotland, which found that those living in the most deprived areas, with multimorbidity, and in urban areas, had lower satisfaction with care received compared to least deprived, without multimorbidity, and in rural areas, respectively. [29] They also augment qualitative work we have conducted evaluating the effects of the new Scottish GP contract [30,31], where multimorbidity and deprivation are identified as key areas where the new GP contract does not appear to be helping. Patients in deprived areas of Scotland have poorer health and more multimorbidity than in more affluent areas, and this is reflected in the GP consultation in terms of having more problems to discuss, and these problems being more complex. [29] Yet previous research has shown that consultations are shorter in deprived areas than affluent, and GPs are more stressed. [32] Giving targeted longer consultations to patients with complex needs in deprived areas leads to better outcomes. [33,34] Although longer consultations for patients with complex problems was a key ambition of the new Scottish GP contract, there is no evidence that this has been achieved. [35]

Furthermore, we corroborate findings from a multinational paper drawing from data across 31 countries, that women and those with lower socioeconomic status have lower satisfaction with general practice, but contradict their findings of no association between satisfaction and age. [36] The temporal reduction in satisfaction we observed mirrors reductions in overall patient experience in England over a similar time period [37] suggesting a UK-wide issue with patient satisfaction in general practice, and our findings regarding rurality confirm a previous study using unlinked HACE data. [38]

### Implications

There are several important implications resulting from our findings. First, the persistent decline in overall satisfaction, access, and (to a lesser extent consultation quality) even after significant reforms in the organisation of general practice in Scotland, suggests that the new Scottish GP contract has not effectively improved patient experiences. The wider context underpinning these findings include the fact that the number of whole-time equivalent GPs in Scotland has been falling over the period of this study,[39] the number of registered patients nationally has been increasing, the number of practices in Scotland has decreased, and patient complexity has increased. [40]

Second, the widening inequalities between the most and least deprived populations, particularly among those with multimorbidity, highlight a failure to reduce health disparities. The contract’s aim to mitigate these growing inequalities has not been realised, and the increasing gap in patient experiences points to an urgent need for targeted strategies to promote equity in healthcare access and outcomes. Recent work in Scotland found no substantial change in the inverse care law over the last twenty years, with a persisting mismatch between patient need and GP supply in deprived areas compared with affluent areas. [41]

Future research should explore the comparative quality of consultations between doctors and other healthcare professionals, such as nurses or physician assistants. As multidisciplinary teams become more integral to primary care, understanding how patient experiences differ across these professionals could inform strategies to optimise care delivery. Additionally, further research could investigate the impact of continuity of care on patient satisfaction and outcomes, particularly in a context where patients may be seeing a variety of healthcare providers rather than a single GP. Finally, the potential long-term impact of remote consultations (e.g., telephone or video appointments) on patient satisfaction and outcomes is uncertain, and research to understand how shifts to remote consultations affects perceptions of access and quality of care is needed as these technologies mature and spread.

## Supporting information

Figure S1 Percentage of positive responses to doctor listened question 2011/12 by A) Age and sex B) SIMD decile C) Number of Health Issues and D) Urban/Rural Class. (DOCX)

Figure S2 Percentage of positive responses to Time with doctor question 2011/12 by A) Age and sex B) SIMD decile C) Number of Health Issues and D) Urban/Rural Class. (DOCX)

Table S1 Overall relative Index on Inequality (RII) between most and least deprived SIMD deciles 2011/12–2021/22. (DOCX)

Figure S3 Overall relative Index on Inequality (RII) between most and least deprived SIMD deciles 2011/12–2021/22with 95% Confidence Interval (CI). (DOCX)

Figure S4 Relative Index of Inequality (RII) and 95% Confidence Interval (CI) for doctor listened question between most a least deprived SIMD decile by A) Age and sex B) Number of Health Issues and c) Urban/Rural Class. (DOCX)

Figure S5 Relative Index of Inequality (RII) and 95% Confidence Interval (CI) for Time with doctor question between most a least deprived SIMD decile by A) Age and sex B) Number of Health Issues and c) Urban/Rural Class. (DOCX)

Table S2 Overall relative Index on Inequality (RII) between most and least deprived SIMD deciles by age and sex 2011/12–2021/22. (DOCX)

Table S3 Overall relative Index on Inequality (RII) between most and least deprived SIMD deciles by multimorbidity status 2011/12–2021/22. (DOCX)

Table S4 Overall relative Index on Inequality (RII) between most and least deprived SIMD deciles by Urban/Rural (UR) status 2011/12–2021/22. (DOCX)
