# Supplementary material for: Patient satisfaction with general practice in Scotland 2011/12 to 2021/22
Source: PLoS One. 2025 Apr 30;20(4):e0322095. doi: 10.1371/journal.pone.0322095 (PMC12043161; doi:10.1371/journal.pone.0322095)
Supplement: Figure S1 — (DOCX) [file pone.0322095.s001.docx]

**Figure S1: Percentage of positive responses to doctor listened question 2011/12 by A) Age and sex B) SIMD decile C) Number of Health Issues and D) Urban/Rural Class**

**
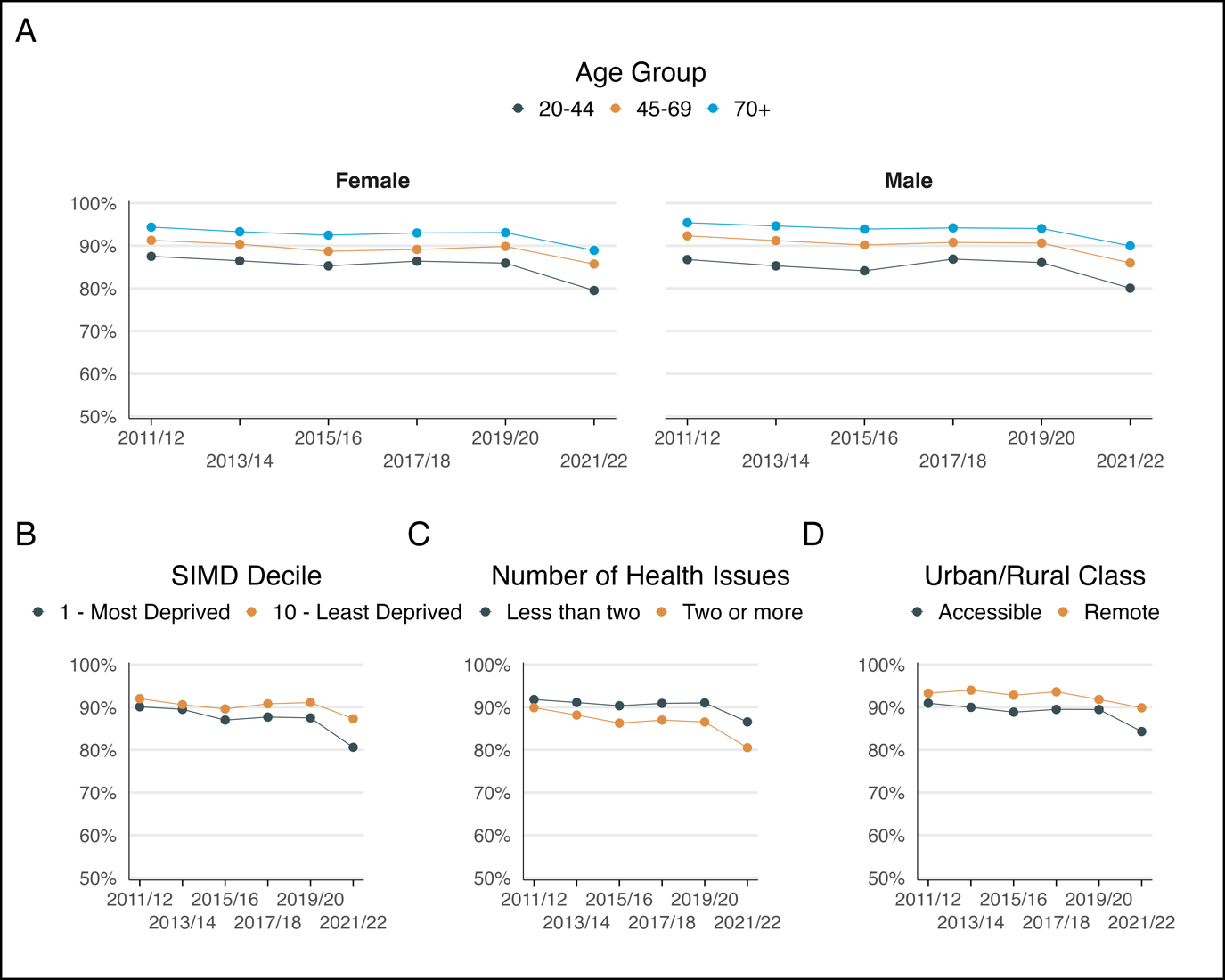
**

**Figure S2: Percentage of positive responses to Time with doctor question 2011/12 by A) Age and sex B) SIMD decile C) Number of Health Issues and D) Urban/Rural Class**

**
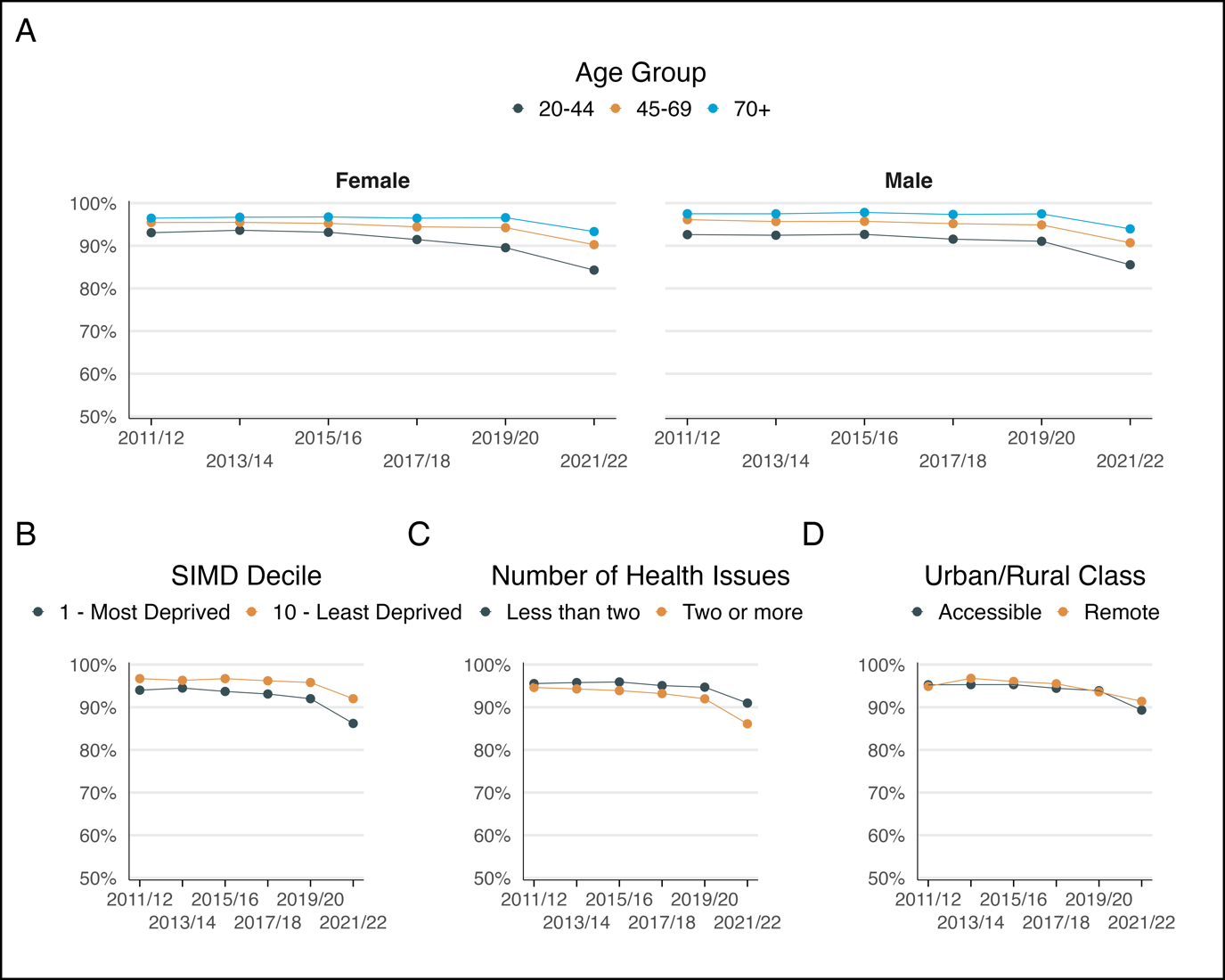
**

**Table S1: Overall relative Index on Inequality (RII) between most and least deprived SIMD deciles 2011/12 to 2021/22**

| **Year** | **Question** | **RII** | **95% CI low** | **95% CI high** |
| --- | --- | --- | --- | --- |
| 2011/12 | Overall Satisfaction | 1.05 | 1.04 | 1.06 |
| 2013/14 | Overall Satisfaction | 1.05 | 1.03 | 1.06 |
| 2015/16 | Overall Satisfaction | 1.06 | 1.04 | 1.07 |
| 2017/18 | Overall Satisfaction | 1.07 | 1.05 | 1.09 |
| 2019/20 | Overall Satisfaction | 1.10 | 1.08 | 1.13 |
| 2021/22 | Overall Satisfaction | 1.12 | 1.08 | 1.15 |
| 2011/12 | Access | 1.09 | 1.06 | 1.11 |
| 2013/14 | Access | 1.06 | 1.04 | 1.08 |
| 2015/16 | Access | 1.07 | 1.05 | 1.10 |
| 2017/18 | Access | 1.10 | 1.07 | 1.14 |
| 2019/20 | Access | 1.08 | 1.05 | 1.11 |
| 2021/22 | Access | 1.05 | 1.02 | 1.08 |
| 2011/12 | Listened | 1.02 | 1.02 | 1.03 |
| 2013/14 | Listened | 1.03 | 1.02 | 1.03 |
| 2015/16 | Listened | 1.04 | 1.03 | 1.04 |
| 2017/18 | Listened | 1.04 | 1.03 | 1.05 |
| 2019/20 | Listened | 1.05 | 1.04 | 1.06 |
| 2021/22 | Listened | 1.06 | 1.05 | 1.08 |
| 2011/12 | Time | 1.02 | 1.01 | 1.03 |
| 2013/14 | Time | 1.02 | 1.01 | 1.03 |
| 2015/16 | Time | 1.03 | 1.02 | 1.04 |
| 2017/18 | Time | 1.04 | 1.03 | 1.05 |
| 2019/20 | Time | 1.04 | 1.03 | 1.06 |
| 2021/22 | Time | 1.08 | 1.06 | 1.11 |

**Figure S3: Overall relative Index on Inequality (RII) between most and least deprived SIMD deciles 2011/12 to 2021/22with 95% Confidence Interval (CI)**

**
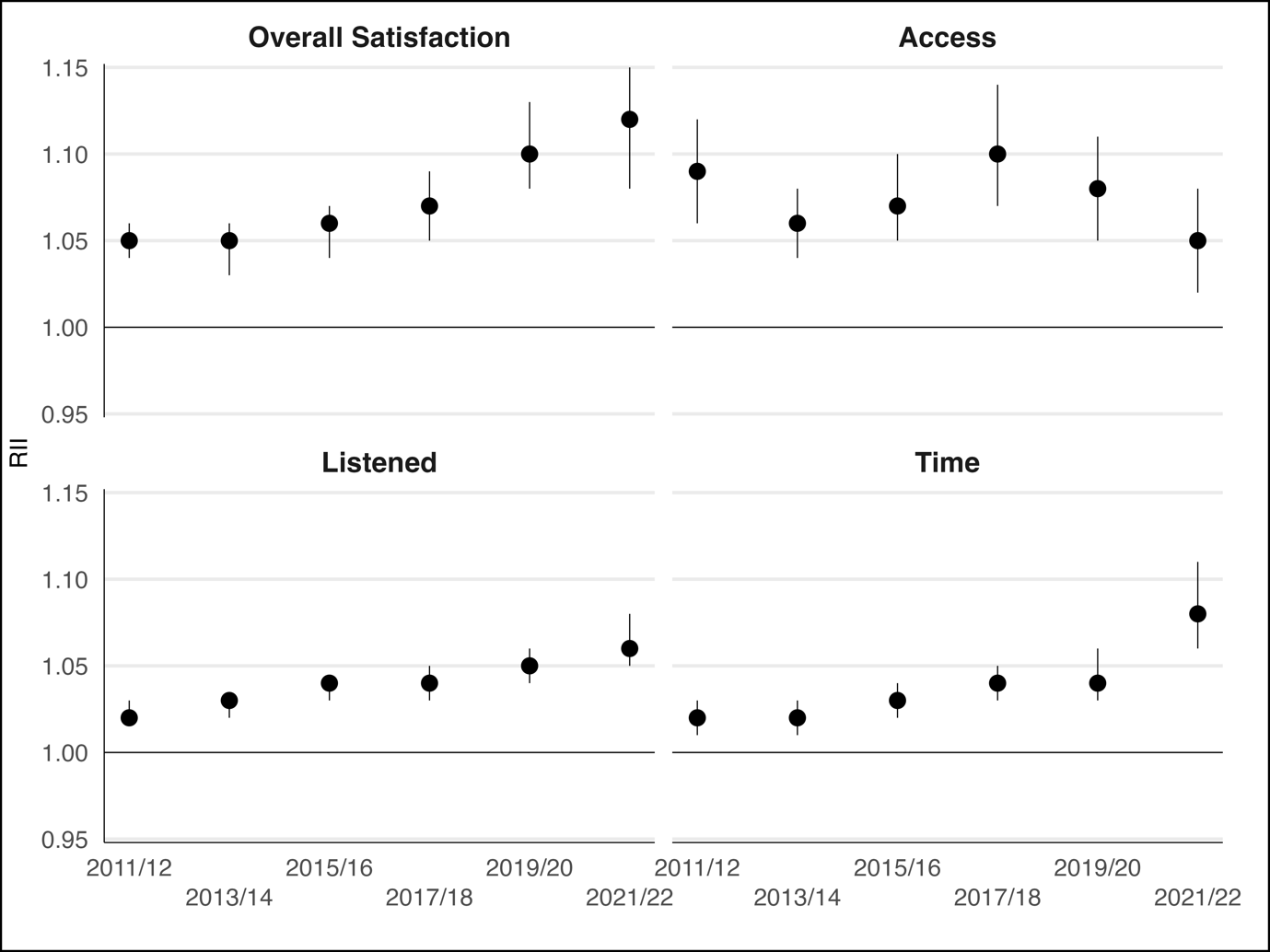
**

**Figure S4: Relative Index of Inequality (RII) and 95% Confidence Interval (CI) for doctor listened question between most a least deprived SIMD decile by A) Age and sex B) Number of Health Issues and c) Urban/Rural Class**

**
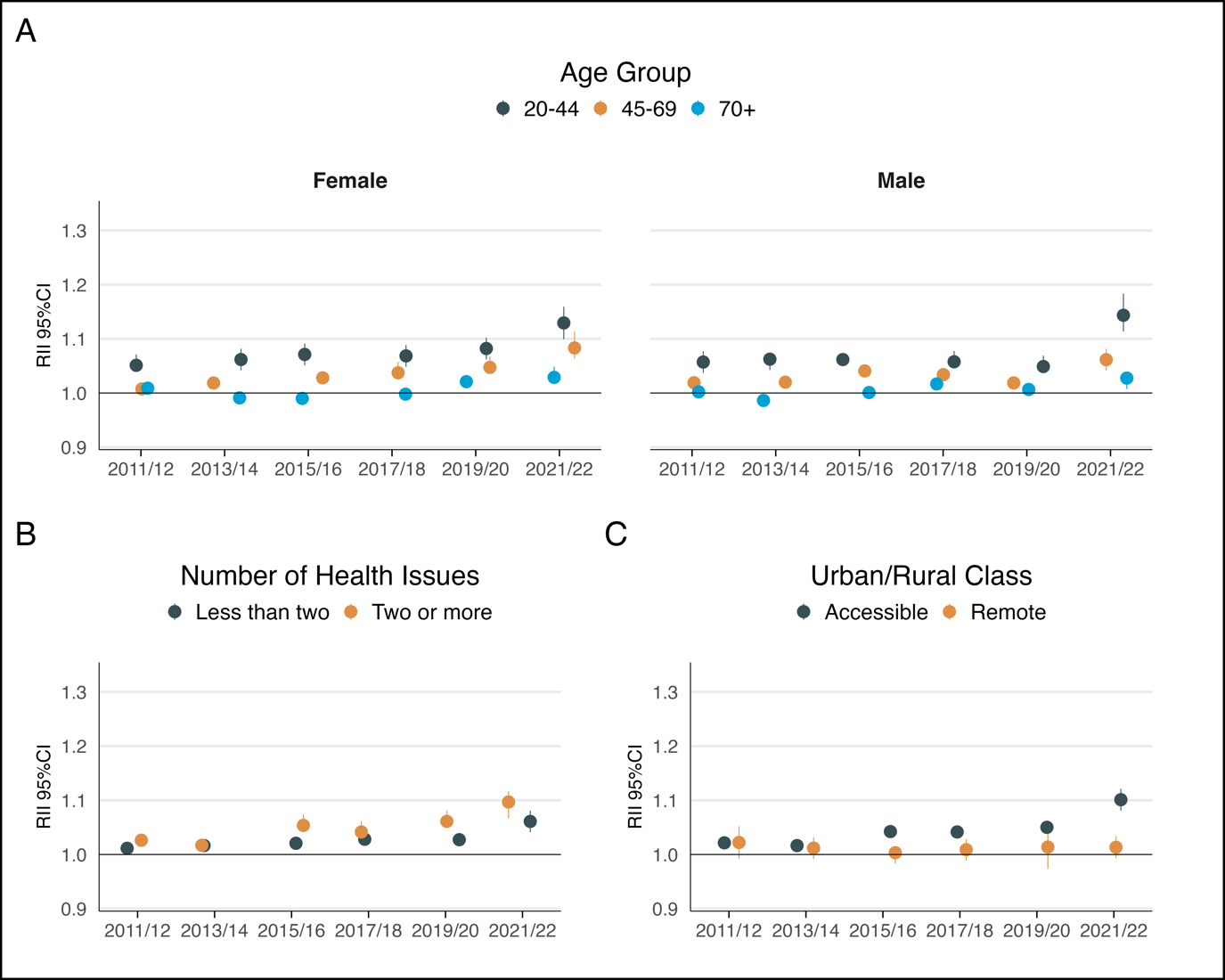
**

**Figure S5: Relative Index of Inequality (RII) and 95% Confidence Interval (CI) for Time with doctor question between most a least deprived SIMD decile by A) Age and sex B) Number of Health Issues and c) Urban/Rural Class**

**
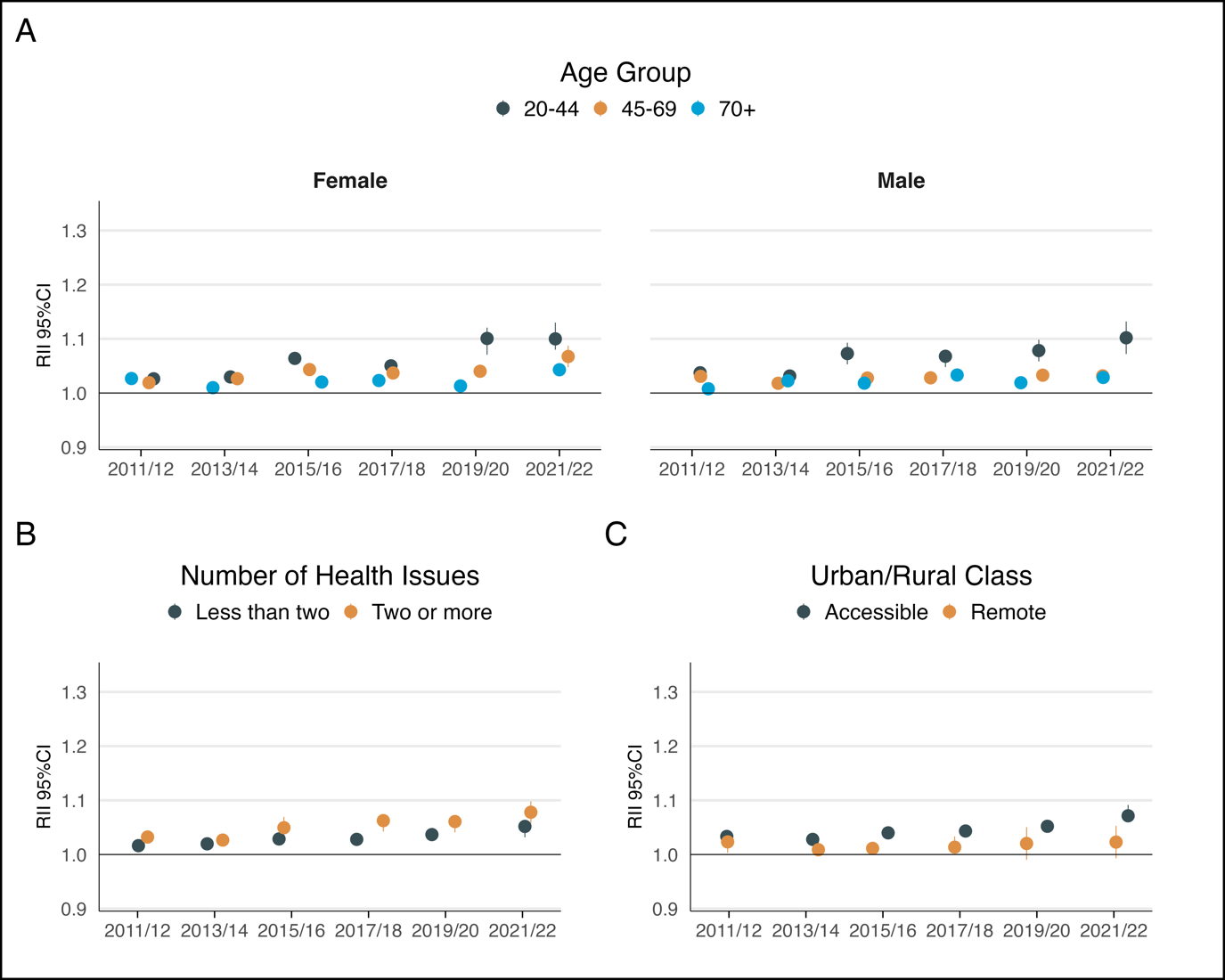
**

**Table S2: Overall relative Index on Inequality (RII) between most and least deprived SIMD deciles by age and sex 2011/12 to 2021/22**

| **Year** | **Question** | **Age band** | **Sex** | **RII** | **95% CI low** | **95% CI high** |
| --- | --- | --- | --- | --- | --- | --- |
| 2011/12 | Overall Satisfaction | 20-44 | Female | 1.11 | 1.08 | 1.13 |
| 2011/12 | Overall Satisfaction | 20-44 | Male | 1.10 | 1.07 | 1.12 |
| 2011/12 | Overall Satisfaction | 45-69 | Female | 1.04 | 1.03 | 1.05 |
| 2011/12 | Overall Satisfaction | 45-69 | Male | 1.03 | 1.02 | 1.04 |
| 2011/12 | Overall Satisfaction | 70+ | Female | 1.04 | 1.03 | 1.05 |
| 2011/12 | Overall Satisfaction | 70+ | Male | 1.01 | 1.01 | 1.02 |
| 2013/14 | Overall Satisfaction | 20-44 | Female | 1.12 | 1.09 | 1.15 |
| 2013/14 | Overall Satisfaction | 20-44 | Male | 1.06 | 1.04 | 1.08 |
| 2013/14 | Overall Satisfaction | 45-69 | Female | 1.06 | 1.04 | 1.07 |
| 2013/14 | Overall Satisfaction | 45-69 | Male | 1.02 | 1.01 | 1.03 |
| 2013/14 | Overall Satisfaction | 70+ | Female | 1.02 | 1.01 | 1.03 |
| 2013/14 | Overall Satisfaction | 70+ | Male | 1.02 | 1.01 | 1.02 |
| 2015/16 | Overall Satisfaction | 20-44 | Female | 1.09 | 1.07 | 1.12 |
| 2015/16 | Overall Satisfaction | 20-44 | Male | 1.09 | 1.06 | 1.11 |
| 2015/16 | Overall Satisfaction | 45-69 | Female | 1.06 | 1.05 | 1.08 |
| 2015/16 | Overall Satisfaction | 45-69 | Male | 1.05 | 1.04 | 1.06 |
| 2015/16 | Overall Satisfaction | 70+ | Female | 1.04 | 1.03 | 1.05 |
| 2015/16 | Overall Satisfaction | 70+ | Male | 1.03 | 1.02 | 1.03 |
| 2017/18 | Overall Satisfaction | 20-44 | Female | 1.14 | 1.10 | 1.17 |
| 2017/18 | Overall Satisfaction | 20-44 | Male | 1.13 | 1.10 | 1.16 |
| 2017/18 | Overall Satisfaction | 45-69 | Female | 1.07 | 1.05 | 1.09 |
| 2017/18 | Overall Satisfaction | 45-69 | Male | 1.04 | 1.03 | 1.05 |
| 2017/18 | Overall Satisfaction | 70+ | Female | 1.06 | 1.04 | 1.07 |
| 2017/18 | Overall Satisfaction | 70+ | Male | 1.05 | 1.04 | 1.07 |
| 2019/20 | Overall Satisfaction | 20-44 | Female | 1.17 | 1.13 | 1.21 |
| 2019/20 | Overall Satisfaction | 20-44 | Male | 1.10 | 1.08 | 1.13 |
| 2019/20 | Overall Satisfaction | 45-69 | Female | 1.12 | 1.09 | 1.15 |
| 2019/20 | Overall Satisfaction | 45-69 | Male | 1.07 | 1.05 | 1.09 |
| 2019/20 | Overall Satisfaction | 70+ | Female | 1.08 | 1.06 | 1.10 |
| 2019/20 | Overall Satisfaction | 70+ | Male | 1.05 | 1.04 | 1.07 |
| 2021/22 | Overall Satisfaction | 20-44 | Female | 1.22 | 1.16 | 1.27 |
| 2021/22 | Overall Satisfaction | 20-44 | Male | 1.20 | 1.15 | 1.25 |
| 2021/22 | Overall Satisfaction | 45-69 | Female | 1.15 | 1.11 | 1.20 |
| 2021/22 | Overall Satisfaction | 45-69 | Male | 1.03 | 1.00 | 1.05 |
| 2021/22 | Overall Satisfaction | 70+ | Female | 1.08 | 1.05 | 1.11 |
| 2021/22 | Overall Satisfaction | 70+ | Male | 1.06 | 1.03 | 1.09 |
| 2011/12 | Access | 20-44 | Female | 1.16 | 1.12 | 1.21 |
| 2011/12 | Access | 20-44 | Male | 1.08 | 1.05 | 1.10 |
| 2011/12 | Access | 45-69 | Female | 1.09 | 1.06 | 1.13 |
| 2011/12 | Access | 45-69 | Male | 1.03 | 1.01 | 1.05 |
| 2011/12 | Access | 70+ | Female | 1.12 | 1.09 | 1.15 |
| 2011/12 | Access | 70+ | Male | 1.06 | 1.04 | 1.08 |
| 2013/14 | Access | 20-44 | Female | 1.13 | 1.09 | 1.17 |
| 2013/14 | Access | 20-44 | Male | 1.05 | 1.03 | 1.06 |
| 2013/14 | Access | 45-69 | Female | 1.09 | 1.05 | 1.12 |
| 2013/14 | Access | 45-69 | Male | 1.01 | 0.99 | 1.03 |
| 2013/14 | Access | 70+ | Female | 1.06 | 1.04 | 1.08 |
| 2013/14 | Access | 70+ | Male | 1.03 | 1.02 | 1.05 |
| 2015/16 | Access | 20-44 | Female | 1.13 | 1.10 | 1.16 |
| 2015/16 | Access | 20-44 | Male | 1.09 | 1.06 | 1.13 |
| 2015/16 | Access | 45-69 | Female | 1.09 | 1.06 | 1.12 |
| 2015/16 | Access | 45-69 | Male | 1.03 | 1.01 | 1.05 |
| 2015/16 | Access | 70+ | Female | 1.07 | 1.04 | 1.10 |
| 2015/16 | Access | 70+ | Male | 1.04 | 1.02 | 1.06 |
| 2017/18 | Access | 20-44 | Female | 1.17 | 1.13 | 1.22 |
| 2017/18 | Access | 20-44 | Male | 1.07 | 1.05 | 1.10 |
| 2017/18 | Access | 45-69 | Female | 1.14 | 1.09 | 1.19 |
| 2017/18 | Access | 45-69 | Male | 1.04 | 1.01 | 1.06 |
| 2017/18 | Access | 70+ | Female | 1.10 | 1.06 | 1.13 |
| 2017/18 | Access | 70+ | Male | 1.07 | 1.04 | 1.10 |
| 2019/20 | Access | 20-44 | Female | 1.12 | 1.09 | 1.15 |
| 2019/20 | Access | 20-44 | Male | 1.09 | 1.06 | 1.12 |
| 2019/20 | Access | 45-69 | Female | 1.10 | 1.06 | 1.14 |
| 2019/20 | Access | 45-69 | Male | 1.04 | 1.02 | 1.07 |
| 2019/20 | Access | 70+ | Female | 1.07 | 1.04 | 1.10 |
| 2019/20 | Access | 70+ | Male | 1.03 | 1.01 | 1.05 |
| 2021/22 | Access | 20-44 | Female | 1.15 | 1.11 | 1.19 |
| 2021/22 | Access | 20-44 | Male | 1.13 | 1.09 | 1.16 |
| 2021/22 | Access | 45-69 | Female | 1.09 | 1.05 | 1.13 |
| 2021/22 | Access | 45-69 | Male | 0.96 | 0.93 | 0.99 |
| 2021/22 | Access | 70+ | Female | 1.03 | 1.01 | 1.06 |
| 2021/22 | Access | 70+ | Male | 0.99 | 0.96 | 1.02 |
| 2011/12 | Listened | 20-44 | Female | 1.03 | 1.03 | 1.04 |
| 2011/12 | Listened | 20-44 | Male | 1.04 | 1.03 | 1.05 |
| 2011/12 | Listened | 45-69 | Female | 1.02 | 1.01 | 1.02 |
| 2011/12 | Listened | 45-69 | Male | 1.03 | 1.02 | 1.03 |
| 2011/12 | Listened | 70+ | Female | 1.03 | 1.02 | 1.03 |
| 2011/12 | Listened | 70+ | Male | 1.01 | 1.00 | 1.01 |
| 2013/14 | Listened | 20-44 | Female | 1.03 | 1.03 | 1.04 |
| 2013/14 | Listened | 20-44 | Male | 1.03 | 1.02 | 1.04 |
| 2013/14 | Listened | 45-69 | Female | 1.03 | 1.02 | 1.04 |
| 2013/14 | Listened | 45-69 | Male | 1.02 | 1.02 | 1.03 |
| 2013/14 | Listened | 70+ | Female | 1.01 | 1.01 | 1.02 |
| 2013/14 | Listened | 70+ | Male | 1.02 | 1.02 | 1.03 |
| 2015/16 | Listened | 20-44 | Female | 1.06 | 1.05 | 1.07 |
| 2015/16 | Listened | 20-44 | Male | 1.07 | 1.05 | 1.09 |
| 2015/16 | Listened | 45-69 | Female | 1.04 | 1.03 | 1.05 |
| 2015/16 | Listened | 45-69 | Male | 1.03 | 1.02 | 1.03 |
| 2015/16 | Listened | 70+ | Female | 1.02 | 1.01 | 1.02 |
| 2015/16 | Listened | 70+ | Male | 1.02 | 1.02 | 1.03 |
| 2017/18 | Listened | 20-44 | Female | 1.05 | 1.04 | 1.06 |
| 2017/18 | Listened | 20-44 | Male | 1.07 | 1.05 | 1.08 |
| 2017/18 | Listened | 45-69 | Female | 1.04 | 1.03 | 1.05 |
| 2017/18 | Listened | 45-69 | Male | 1.03 | 1.02 | 1.04 |
| 2017/18 | Listened | 70+ | Female | 1.02 | 1.02 | 1.03 |
| 2017/18 | Listened | 70+ | Male | 1.03 | 1.02 | 1.03 |
| 2019/20 | Listened | 20-44 | Female | 1.10 | 1.07 | 1.12 |
| 2019/20 | Listened | 20-44 | Male | 1.08 | 1.06 | 1.10 |
| 2019/20 | Listened | 45-69 | Female | 1.04 | 1.03 | 1.05 |
| 2019/20 | Listened | 45-69 | Male | 1.03 | 1.02 | 1.04 |
| 2019/20 | Listened | 70+ | Female | 1.01 | 1.01 | 1.02 |
| 2019/20 | Listened | 70+ | Male | 1.02 | 1.02 | 1.03 |
| 2021/22 | Listened | 20-44 | Female | 1.10 | 1.08 | 1.13 |
| 2021/22 | Listened | 20-44 | Male | 1.10 | 1.07 | 1.13 |
| 2021/22 | Listened | 45-69 | Female | 1.07 | 1.05 | 1.09 |
| 2021/22 | Listened | 45-69 | Male | 1.03 | 1.02 | 1.04 |
| 2021/22 | Listened | 70+ | Female | 1.04 | 1.03 | 1.05 |
| 2021/22 | Listened | 70+ | Male | 1.03 | 1.02 | 1.04 |
| 2011/12 | Time | 20-44 | Female | 1.05 | 1.04 | 1.07 |
| 2011/12 | Time | 20-44 | Male | 1.06 | 1.04 | 1.08 |
| 2011/12 | Time | 45-69 | Female | 1.01 | 1.00 | 1.01 |
| 2011/12 | Time | 45-69 | Male | 1.02 | 1.02 | 1.03 |
| 2011/12 | Time | 70+ | Female | 1.01 | 1.00 | 1.01 |
| 2011/12 | Time | 70+ | Male | 1.00 | 1.00 | 1.01 |
| 2013/14 | Time | 20-44 | Female | 1.06 | 1.04 | 1.08 |
| 2013/14 | Time | 20-44 | Male | 1.06 | 1.04 | 1.07 |
| 2013/14 | Time | 45-69 | Female | 1.02 | 1.01 | 1.03 |
| 2013/14 | Time | 45-69 | Male | 1.02 | 1.01 | 1.03 |
| 2013/14 | Time | 70+ | Female | 0.99 | 0.98 | 0.99 |
| 2013/14 | Time | 70+ | Male | 0.99 | 0.99 | 1.00 |
| 2015/16 | Time | 20-44 | Female | 1.07 | 1.05 | 1.09 |
| 2015/16 | Time | 20-44 | Male | 1.06 | 1.05 | 1.07 |
| 2015/16 | Time | 45-69 | Female | 1.03 | 1.02 | 1.04 |
| 2015/16 | Time | 45-69 | Male | 1.04 | 1.03 | 1.05 |
| 2015/16 | Time | 70+ | Female | 0.99 | 0.98 | 1.00 |
| 2015/16 | Time | 70+ | Male | 1.00 | 0.99 | 1.01 |
| 2017/18 | Time | 20-44 | Female | 1.07 | 1.05 | 1.09 |
| 2017/18 | Time | 20-44 | Male | 1.06 | 1.05 | 1.08 |
| 2017/18 | Time | 45-69 | Female | 1.04 | 1.03 | 1.06 |
| 2017/18 | Time | 45-69 | Male | 1.03 | 1.02 | 1.04 |
| 2017/18 | Time | 70+ | Female | 1.00 | 0.99 | 1.01 |
| 2017/18 | Time | 70+ | Male | 1.02 | 1.01 | 1.02 |
| 2019/20 | Time | 20-44 | Female | 1.08 | 1.06 | 1.10 |
| 2019/20 | Time | 20-44 | Male | 1.05 | 1.04 | 1.07 |
| 2019/20 | Time | 45-69 | Female | 1.05 | 1.04 | 1.07 |
| 2019/20 | Time | 45-69 | Male | 1.02 | 1.01 | 1.03 |
| 2019/20 | Time | 70+ | Female | 1.02 | 1.01 | 1.03 |
| 2019/20 | Time | 70+ | Male | 1.01 | 1.01 | 1.02 |
| 2021/22 | Time | 20-44 | Female | 1.13 | 1.10 | 1.16 |
| 2021/22 | Time | 20-44 | Male | 1.14 | 1.11 | 1.18 |
| 2021/22 | Time | 45-69 | Female | 1.08 | 1.06 | 1.11 |
| 2021/22 | Time | 45-69 | Male | 1.06 | 1.04 | 1.08 |
| 2021/22 | Time | 70+ | Female | 1.03 | 1.02 | 1.05 |
| 2021/22 | Time | 70+ | Male | 1.03 | 1.01 | 1.04 |

**Table S3: Overall relative Index on Inequality (RII) between most and least deprived SIMD deciles by multimorbidity status 2011/12 to 2021/22**

| **Year** | **Question** | **N conditions** | **RII** | **Rii 95% CI low** | **RII 95% CI high** |
| --- | --- | --- | --- | --- | --- |
| 2011/12 | Overall Satisfaction | Less than two | 1.04 | 1.03 | 1.06 |
| 2011/12 | Overall Satisfaction | Two or more | 1.06 | 1.05 | 1.07 |
| 2013/14 | Overall Satisfaction | Less than two | 1.04 | 1.03 | 1.06 |
| 2013/14 | Overall Satisfaction | Two or more | 1.05 | 1.04 | 1.06 |
| 2015/16 | Overall Satisfaction | Less than two | 1.05 | 1.04 | 1.06 |
| 2015/16 | Overall Satisfaction | Two or more | 1.06 | 1.05 | 1.07 |
| 2017/18 | Overall Satisfaction | Less than two | 1.07 | 1.05 | 1.08 |
| 2017/18 | Overall Satisfaction | Two or more | 1.07 | 1.06 | 1.09 |
| 2019/20 | Overall Satisfaction | Less than two | 1.09 | 1.06 | 1.11 |
| 2019/20 | Overall Satisfaction | Two or more | 1.11 | 1.08 | 1.14 |
| 2021/22 | Overall Satisfaction | Less than two | 1.09 | 1.06 | 1.12 |
| 2021/22 | Overall Satisfaction | Two or more | 1.13 | 1.09 | 1.17 |
| 2011/12 | Access | Less than two | 1.08 | 1.05 | 1.11 |
| 2011/12 | Access | Two or more | 1.12 | 1.09 | 1.14 |
| 2013/14 | Access | Less than two | 1.06 | 1.03 | 1.08 |
| 2013/14 | Access | Two or more | 1.06 | 1.04 | 1.08 |
| 2015/16 | Access | Less than two | 1.06 | 1.03 | 1.08 |
| 2015/16 | Access | Two or more | 1.13 | 1.09 | 1.16 |
| 2017/18 | Access | Less than two | 1.09 | 1.05 | 1.12 |
| 2017/18 | Access | Two or more | 1.12 | 1.09 | 1.16 |
| 2019/20 | Access | Less than two | 1.06 | 1.03 | 1.09 |
| 2019/20 | Access | Two or more | 1.09 | 1.06 | 1.12 |
| 2021/22 | Access | Less than two | 1.03 | 1.00 | 1.05 |
| 2021/22 | Access | Two or more | 1.06 | 1.02 | 1.10 |
| 2011/12 | Listened | Less than two | 1.02 | 1.01 | 1.02 |
| 2011/12 | Listened | Two or more | 1.03 | 1.02 | 1.04 |
| 2013/14 | Listened | Less than two | 1.02 | 1.02 | 1.03 |
| 2013/14 | Listened | Two or more | 1.03 | 1.02 | 1.04 |
| 2015/16 | Listened | Less than two | 1.03 | 1.02 | 1.03 |
| 2015/16 | Listened | Two or more | 1.05 | 1.04 | 1.07 |
| 2017/18 | Listened | Less than two | 1.03 | 1.02 | 1.04 |
| 2017/18 | Listened | Two or more | 1.06 | 1.04 | 1.07 |
| 2019/20 | Listened | Less than two | 1.04 | 1.03 | 1.05 |
| 2019/20 | Listened | Two or more | 1.06 | 1.04 | 1.07 |
| 2021/22 | Listened | Less than two | 1.05 | 1.03 | 1.06 |
| 2021/22 | Listened | Two or more | 1.08 | 1.06 | 1.10 |
| 2011/12 | Time | Less than two | 1.01 | 1.01 | 1.02 |
| 2011/12 | Time | Two or more | 1.03 | 1.02 | 1.04 |
| 2013/14 | Time | Less than two | 1.02 | 1.01 | 1.02 |
| 2013/14 | Time | Two or more | 1.02 | 1.01 | 1.03 |
| 2015/16 | Time | Less than two | 1.02 | 1.01 | 1.02 |
| 2015/16 | Time | Two or more | 1.05 | 1.04 | 1.07 |
| 2017/18 | Time | Less than two | 1.03 | 1.02 | 1.04 |
| 2017/18 | Time | Two or more | 1.04 | 1.03 | 1.06 |
| 2019/20 | Time | Less than two | 1.03 | 1.02 | 1.04 |
| 2019/20 | Time | Two or more | 1.06 | 1.05 | 1.08 |
| 2021/22 | Time | Less than two | 1.06 | 1.04 | 1.08 |
| 2021/22 | Time | Two or more | 1.10 | 1.07 | 1.12 |

**Table S4: Overall relative Index on Inequality (RII) between most and least deprived SIMD deciles by Urban/Rural (UR) status 2011/12 to 2021/22**

| **Year** | **Question** | **UR status** | **RII** | **95% CI low** | **95% CI high** |
| --- | --- | --- | --- | --- | --- |
| 2011/12 | Overall Satisfaction | Accessible | 1.05 | 1.04 | 1.06 |
| 2011/12 | Overall Satisfaction | Remote | 1.03 | 1.00 | 1.07 |
| 2013/14 | Overall Satisfaction | Accessible | 1.05 | 1.04 | 1.06 |
| 2013/14 | Overall Satisfaction | Remote | 1.03 | 1.01 | 1.06 |
| 2015/16 | Overall Satisfaction | Accessible | 1.06 | 1.05 | 1.08 |
| 2015/16 | Overall Satisfaction | Remote | 1.03 | 0.99 | 1.06 |
| 2017/18 | Overall Satisfaction | Accessible | 1.08 | 1.06 | 1.10 |
| 2017/18 | Overall Satisfaction | Remote | 1.05 | 1.00 | 1.10 |
| 2019/20 | Overall Satisfaction | Accessible | 1.11 | 1.09 | 1.14 |
| 2019/20 | Overall Satisfaction | Remote | 1.03 | 0.98 | 1.09 |
| 2021/22 | Overall Satisfaction | Accessible | 1.14 | 1.11 | 1.18 |
| 2021/22 | Overall Satisfaction | Remote | 1.01 | 0.96 | 1.07 |
| 2011/12 | Access | Accessible | 1.09 | 1.07 | 1.11 |
| 2011/12 | Access | Remote | 1.06 | 0.97 | 1.16 |
| 2013/14 | Access | Accessible | 1.06 | 1.05 | 1.08 |
| 2013/14 | Access | Remote | 1.06 | 0.98 | 1.14 |
| 2015/16 | Access | Accessible | 1.08 | 1.06 | 1.11 |
| 2015/16 | Access | Remote | 1.02 | 0.95 | 1.09 |
| 2017/18 | Access | Accessible | 1.11 | 1.09 | 1.14 |
| 2017/18 | Access | Remote | 1.04 | 0.95 | 1.15 |
| 2019/20 | Access | Accessible | 1.09 | 1.07 | 1.11 |
| 2019/20 | Access | Remote | 1.00 | 0.91 | 1.10 |
| 2021/22 | Access | Accessible | 1.07 | 1.05 | 1.09 |
| 2021/22 | Access | Remote | 0.99 | 0.92 | 1.06 |
| 2011/12 | Listened | Accessible | 1.03 | 1.02 | 1.03 |
| 2011/12 | Listened | Remote | 1.02 | 1.00 | 1.03 |
| 2013/14 | Listened | Accessible | 1.03 | 1.02 | 1.03 |
| 2013/14 | Listened | Remote | 1.01 | 1.00 | 1.03 |
| 2015/16 | Listened | Accessible | 1.04 | 1.03 | 1.05 |
| 2015/16 | Listened | Remote | 1.01 | 1.00 | 1.02 |
| 2017/18 | Listened | Accessible | 1.04 | 1.03 | 1.05 |
| 2017/18 | Listened | Remote | 1.01 | 1.00 | 1.03 |
| 2019/20 | Listened | Accessible | 1.05 | 1.04 | 1.06 |
| 2019/20 | Listened | Remote | 1.02 | 0.99 | 1.05 |
| 2021/22 | Listened | Accessible | 1.07 | 1.06 | 1.09 |
| 2021/22 | Listened | Remote | 1.02 | 0.99 | 1.05 |
| 2011/12 | Time | Accessible | 1.02 | 1.02 | 1.03 |
| 2011/12 | Time | Remote | 1.02 | 0.99 | 1.05 |
| 2013/14 | Time | Accessible | 1.02 | 1.02 | 1.03 |
| 2013/14 | Time | Remote | 1.01 | 0.99 | 1.03 |
| 2015/16 | Time | Accessible | 1.04 | 1.03 | 1.05 |
| 2015/16 | Time | Remote | 1.00 | 0.98 | 1.01 |
| 2017/18 | Time | Accessible | 1.04 | 1.03 | 1.05 |
| 2017/18 | Time | Remote | 1.01 | 0.99 | 1.03 |
| 2019/20 | Time | Accessible | 1.05 | 1.04 | 1.06 |
| 2019/20 | Time | Remote | 1.01 | 0.97 | 1.05 |
| 2021/22 | Time | Accessible | 1.10 | 1.08 | 1.12 |
| 2021/22 | Time | Remote | 1.01 | 0.99 | 1.03 |
